# NlpI-Prc Proteolytic Complex Mediates Peptidoglycan Synthesis and Degradation via Regulation of Hydrolases and Synthases in *Escherichia coli*

**DOI:** 10.3390/ijms242216355

**Published:** 2023-11-15

**Authors:** Xinwei Liu, Tanneke den Blaauwen

**Affiliations:** Bacterial Cell Biology and Physiology, Swammerdam Institute for Life Science, University of Amsterdam, 1098 XH Amsterdam, The Netherlands

**Keywords:** *E. coli*, peptidoglycan, endopeptidase, lytic glycosidase, periplasmic protease, proteolytic control

## Abstract

Balancing peptidoglycan (PG) synthesis and degradation with precision is essential for bacterial growth, yet our comprehension of this intricate process remains limited. The NlpI-Prc proteolytic complex plays a crucial but poorly understood role in the regulation of multiple enzymes involved in PG metabolism. In this paper, through fluorescent D-amino acid 7-hydroxycoumarincarbonylamino-D-alanine (HADA) labeling and immunolabeling assays, we have demonstrated that the NlpI-Prc complex regulates the activity of PG transpeptidases and subcellular localization of PBP3 under certain growth conditions. PBP7 (a PG hydrolase) and MltD (a lytic transglycosylase) were confirmed to be negatively regulated by the NlpI-Prc complex by an in vivo degradation assay. The endopeptidases, MepS, MepM, and MepH, have consistently been demonstrated as redundantly essential “space makers” for nascent PG insertion. However, we observed that the absence of NlpI-Prc complex can alleviate the lethality of the *mepS mepM mepH* mutant. A function of PG lytic transglycosylases MltA and MltD as “space makers” was proposed through multiple gene deletions. These findings unveil novel roles for NlpI-Prc in the regulation of both PG synthesis and degradation, shedding light on the previously undiscovered function of lytic transglycosylases as “space makers” in PG expansion.

## 1. Introduction

Peptidoglycan (PG), a net-like polymer provides structural support and protects bacterial cells from osmotic pressure [1]. The PG consists of alternating *N*-acetylglucosamine (NAG) and *N*-acetylmuramic acid (NAM) glycan strands crosslinked by short peptides [2]. In Gram-negative bacteria, the peptide is made of the amino-acid residues, L-alanine, D-glutamic acid, meso-diaminopimelic acid (Dap), and two D-alanines. Approximately, 40% of the neighbouring short peptides are crosslinked to each other, creating a mesh-like macromolecule that gives PG its stiffness [3]. However, PG exhibits both rigid and dynamic characteristics during bacterial growth. It undergoes continuous synthesis and remodelling processes to accommodate bacterial growth, division, and environmental changes [3,4].

For the synthesis of new PG, glycosyltransferases (GTases) are required to polymerize the glycan strands, while transpeptidases (TPases) are responsible for establishing D-Ala-Dap (4-3) or Dap-Dap (3-3) crosslinks [5]. Class A penicillin-binding proteins (PBPs) PBP1A and PBP1B play an important role in catalysing both glycosylation and transpeptidation reactions [6,7,8]. They are essential for bacterial survival, but the loss of one of them does not result in lethality [9,10]. Class B PBPs (PBP2 and PBP3) are monofunctional TPases [11,12,13]. The elongasome and divisome are multi-protein complexes involved in the insertion of nascent peptidoglycan (PG) into the existing PG layers [14,15,16]. In the elongasome, RodA provides GTase activity and PBP2 is the main DD-transpeptidase that introduces 4-3 crosslinks between the short peptide chains [12,17]. Under the ultimate control of the tubulin homologue FtsZ, the divisome promotes the synthesis of septal PG at the division site [15,18]. In this complex, the GTase and TPase couple FtsW and PBP3 together with PBP1B provide the new PG synthesis activity [11,19]. PBP3 and the transpeptidase activity of PBP1B are depressed until enough FtsN accumulates at the division site [20,21].

The insertion of new peptidoglycan units into the existing layer is essential for PG expansion and bacterial integrity. Due to the net-like structure of PG, hydrolytic enzymes have been predicted to be essential for the insertion of nascent PG [3,22,23]. In *E. coli*, hydrolytic endopeptidases, carboxypeptidases, amidases, and lytic transglycosylases are involved in peptidoglycan metabolism [24]. In 2012, Singh et al. proved that endopeptidases are vital for nascent PG insertion into the existing PG structure [25]. Endopeptidases cleave the crosslinked bond of the peptides that bridge the glycan strands. In *E. coli*, eight hydrolytic enzymes exhibit endopeptidase activity. Among these, endopeptidases MepM, MepS, and MepH were found to be redundantly essential for peptidoglycan expansion and tightly regulated during cell growth [25]. These three endopeptidases were regarded as “space-makers” aiding the insertion of nascent PG [26]. Meanwhile, it is crucial that the cell exercises careful control over these hydrolytic enzymes to avoid anomalous hydrolysis and preserve cell wall integrity. This requires a delicate balance between PG synthesis and hydrolysis.

The periplasmic protease Prc and the lipoprotein NlpI are involved in the negative regulation of endopeptidases, including MepS and MepH [27,28]. NlpI directly interacts with MepS and facilitates the degradation of MepS by Prc [28,29]. In addition to the endopeptidases, it has been discovered that the NlpI-Prc protease complex negatively regulates the transglycosylases, MltB, DigH, and MltG [30,31]. Furthermore, the NlpI-Prc complex has an impact on several proteins involved in PG synthesis, such as PBP1A, PBP1B, and PBP3 [32,33]. Prc cleaves the C-terminal 11 residue peptide of PBP3 to generate the mature protein [33,34,35].

Lytic transglycosylases have a crucial function in cleaving the glycan strands within peptidoglycan. The genome of *E. coli* encodes eight lytic transglycosylases, including six outer membrane-bound lytic transglycosylases (LTs) designated MltA to MltF, and a soluble periplasmic LT called Slt70 [36,37,38,39]. In addition, a division-specific glycosyl hydrolase DigH has been described, which plays a role in cell separation by specifically cleaving glycan strands lacking stem peptides at the septum [30]. Previous studies have confirmed several fundamental functions of lytic transglycosylases, including the release of nascent peptidoglycan, separation of daughter cells, and peptidoglycan recycling [30,31,40]. In the model of peptidoglycan expansion, the role of endopeptidases as “space-makers” has been emphasized. However, less is known about the potential “space-makers” activity of lytic transglycosylases. The average chain length of the glycan strands was found to be 21 disaccharide units, ranging from 2 to 30 disaccharide units [41]. In theory, the cleavage of peptidoglycan strands by lytic transglycosylases, as well as the removal of shorter PG strands, can lead to an increase in available space within the cell wall and allow the insertion of newly synthesized material.

The delicate balance between PG synthesis and its degradation is crucial for bacterial growth. The NlpI-Prc proteolytic complex plays an important but less well-known role in this process [32,42,43]. In this paper, the absence of NlpI-Prc is proven to affect the activity of transpeptidases for PG synthesis and PBP3 was found to lose its midcell localization in the Δ*nlpI* strain cultured in LB medium without NaCl at 42 °C. In addition, new substrates (PBP7 and MltD) for the NlpI-Prc proteolytic complex were confirmed. A novel function of lytic transglycosylases as “space makers” in PG expansion is proposed in this work. These findings highlight the importance of the regulatory role of NlpI-Prc in PG synthesis and degradation and reveal the “space-makers” function of lytic transglycosylases.

## 2. Results

### 2.1. PBPs Abundance Changes in E. coli Mutants Lacking NlpI, Prc, or Both

**Figure 1:**
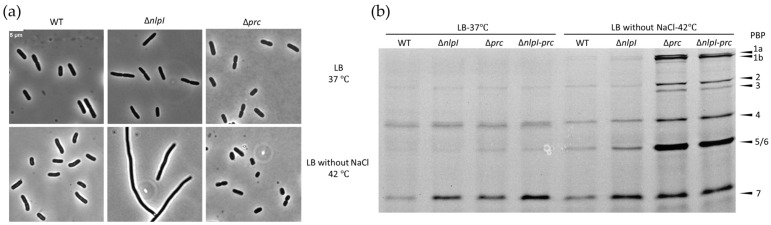
Abundance changes in PBPs in Δ*nlpI*, Δ*prc,* and Δ*nlpI*-*prc* mutant strains: (**a**) Phase-contrast images of WT and mutant strains. The strains were initially cultured in LB medium for 3 h from overnight cultures. After washing two times with LB medium without NaCl, strains were incubated in LB medium with or without NaCl at temperatures of 37 °C and 42 °C for an additional 2 h. The cultures were fixed with 2.8% formaldehyde and 0.04% glutaraldehyde (FA/GA) and imaged using phase-contrast microscopy. The scale bar equals 5 µm and (**b**) Bocillin-FL binding to penicillin-binding proteins (PBPs). Strains cultured as indicated were washed twice with PBS and incubated with PBS containing Bocillin-FL for 10 min at room temperature (RT). The PBPs of different strains were visualized by a 10% SDS-PAGE. The abundance of PBPs in WT cultured in different conditions serve as control for mutans cultured in the same condition.

The NlpI-Prc system plays a crucial role in mediating PG synthesis and remodelling processes in *E. coli.* Consistent with previous findings [33,42], the absence of NlpI in *E. coli* leads to cell filamentation in LB medium without NaCl at 42 °C (Figure 1a), and Prc is essential for cell survival and growth under the same conditions (Figure S1). This observation indicates the importance of NlpI-Prc in maintaining cell growth and morphology of *E. coli* under these specific growth conditions. Previous studies have primarily focused on investigating the regulation of hydrolytic enzymes by the NlpI-Prc complex.

To examine the potential impact of NlpI-Prc on the abundance of PBPs involved in PG synthesis and remodelling, we conducted Bocillin FL-binding assays on different strains grown in LB medium with and without NaCl at temperatures of 37 and 42 °C. Bocillin FL, a fluorescent analogue of penicillin V, was employed to label a wide range of PBPs [44].

From Figure 1b and Figure S1b, it can be observed that, in LB medium at 37 °C, the abundance of most PBPs remained relatively unchanged in the absence of NlpI and Prc, with the exception of PBP5 and PBP7. Compared to wild-type (WT) cells, PBP7 in Δ*prc* exhibited a noticeable increase in abundance under the given conditions, whereas PBP5 showed a slight increase. When cells were grown in LB medium lacking NaCl at 42 °C, the absence of Prc caused the accumulation of all PBPs. This suggests that the growth arrest of Δ*prc* in LB medium without NaCl at 42 °C is not caused by the absence of PBPs. On the contrary, the PBPs are seemingly more abundant in Δ*prc*, which might be due to protein synthesis not immediately halted upon growth cessation in Δ*prc*.

NlpI protects PBP3 from cleavage by Prc [45], and the filamentation observed in the Δ*nlpI* strain cultured in LB medium without NaCl at 42 °C raised the question of whether the absence of PBP3 contributes to the filamentous phenotype. To overcome the limitations of detecting PBP3 in Bocillin-FL binding assays, we conducted an analysis of PBP3 abundance using an affinity purified PBP3 antibody [46]. This allowed us to investigate the abundance of PBP3 in different strains cultured in LB medium with and without NaCl at temperatures of 37 and 42 °C. As shown in Figure S1c, the abundance of PBP3 was not reduced in Δ*nlpI* strain cultured in LB medium without NaCl at 42 °C compared to WT.

### 2.2. NlpI Influences Subcellular Localization of PBP3

**Figure 2:**
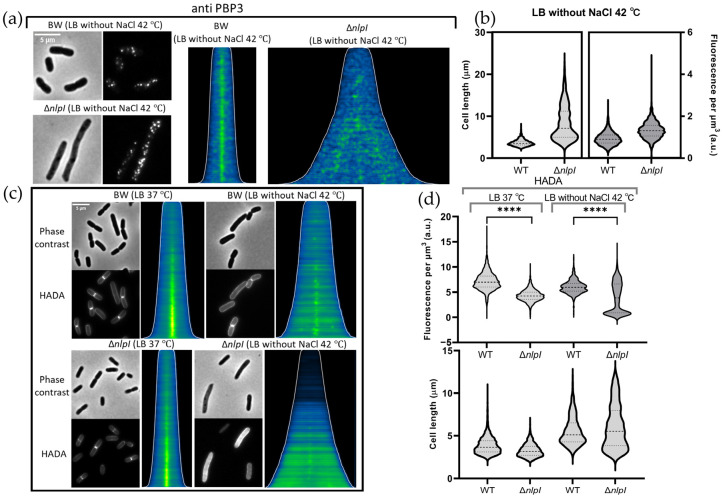
Midcell localization of PBP3 is undermined and the activity of transpeptidases is weaker in Δ*nlpI* cultured in LB medium without NaCl at 42 °C compared with wild-type (BW25113) cells cultured in the same condition: (**a**) Cells of both WT and Δ*nlpI* strains, cultured in LB medium without NaCl for 1 h, were fixed using FA/GA and subjected to immunolabeling using antibodies specific to PBP3. Phase-contrast images, corresponding fluorescence images, and demographs showing the fluorescence localization of PBP3, with cells sorted according to their cell length. The scale bar equals 5 µm. The numbers of cells analysed were 2822 and 1450 for WT and Δ*nlpI*, respectively; (**b**) The cell length and fluorescence per µm^3^ of WT and Δ*nlpI* strains, immunolabeled with PBP3 antibody; (**c**) Phase-contrast and fluorescence images of cells cultured in LB medium with or without NaCl containing 250 μM HADA at 37 and 42 °C. The scale bar equals 5 µm. Demographs of the concentration of HADA in the strains sorted according to cell length. The white line indicates the length of the cells. The number of cells analysed were 2235 and 504 for WT, 2636 and 993 for Δ*nlpI* grown in LB medium at 37 °C and LB medium without NaCl at 42 °C, respectively; and (**d**) The cell length and fluorescence per µm^3^ of HADA in WT and Δ*nlpI* cultured in LB medium with or without NaCl at 37 and 42 °C. The fluorescence concentration of WT cells cultured in different conditions serve as control for the Δ*nlpI* strain cultures in the same condition. Statistical significance, determined through unpaired *t*-test, was indicated as **** *p* ≤ 0.0001.

Based on the observation that Δ*nlpI* is able to grow and form smooth filaments in LB medium without NaCl at 42 °C, we speculate that the divisome may be dysfunctional (Figure 1a and Figure S1). PBP3 is a crucial protein of the divisome that is protected from Prc cleavage by NlpI [33,45]. A potential interaction between NlpI and PBP3 was observed [32,45]. The subcellular localization of PBP3 was investigated in the Δ*nlpI* mutant. PBP3 was found to have lost its characteristic midcell localization in the Δ*nlpI* strain cultured in LB medium without NaCl at 42 °C (Figure 2a). Consistent with previous findings, the abundance of PBP3 was not decreased in the Δ*nlpI* strain cultured in LB medium without NaCl at 42 °C (Figure 2b).

The fluorescent D-amino acid 7-hydroxycoumarincarbonylamino-D-alanine (HADA) can be incorporated into PG by transpeptidases and used as a tool for the assessment of new PG synthesis [47,48,49]. As shown in Figure 2c, the transpeptidase activity in the divisome of Δ*nlpI* cells cultured in LB medium without NaCl at 42 °C is significantly reduced (Figure 2c,d). The loss of PBP3 mid-localization in the Δ*nlpI* mutant is consistent with the observed reduction in divisome transpeptidase activity.

### 2.3. The Absence of Prc Reduces the Activity of Some Transpeptidases

Prc is vital for cell growth under high-temperature conditions in hypotonic media [33]. The abundance of crucial transpeptidases, including PBP1A, PBP1B, PBP2, and PBP3, investigated by Bocillin-FL in Δ*prc* cultured in LB medium without NaCl at 42 °C did not decrease but showed rather an increase (Figure 1b). The growth restriction of Δ*prc* is therefore not caused by absence of crucial transpeptidases. The activity of the transpeptidases in Δ*prc* was examined by the fluorescent D-amino acid HADA.

**Figure 3:**
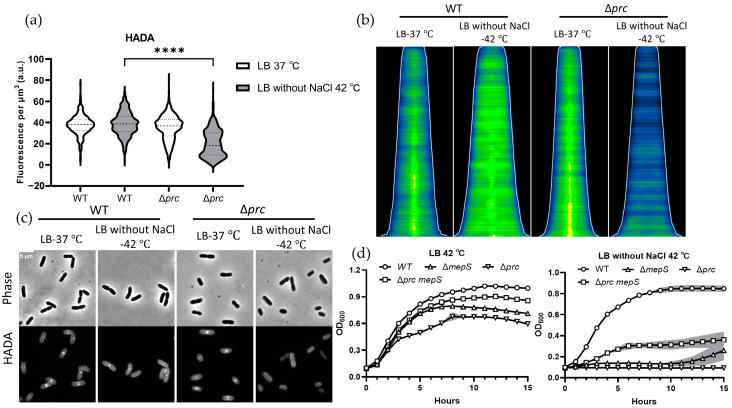
The activity of transpeptidases is weaker in Δ*prc* cultured in LB medium without NaCl at 42 °C compared with wild-type (BW25113) cells cultured in the same condition: (**a**) HADA incorporation in WT and Δ*prc* cultured in LB at 37 °C and LB without NaCl at 42 °C. Values in violin bar graphs represent mean fluorescence concentration quantified from more than 300 cells. The fluorescence concentration of WT cultured in different conditions serve as control for Δ*prc* cultured in the same condition. Statistical significance determined using an unpaired *t* test, was indicated as **** *p* ≤ 0.0001; (**b**) Demographs of the concentration of HADA in the strains sorted according to cell length. The white line indicates the length of the cells The number of cells analysed were 315 and 364 for WT, 814 and 530 for Δ*nlpI*, for the cells grown in LB medium at 37 °C and LB medium without NaCl at 42 °C, respectively; (**c**) Phase-contrast and fluorescence images of cells cultured in medium containing 250 μM HADA. The scale bar equals 5 µm; and (**d**) Growth curve of strains in LB medium with or without NaCl at 42 °C. The growth curve of WT cultured in different conditions serve as positive control for mutants cultured in the same condition. The growth curves were performed in triplicate for each mutant. The solid lines and their corresponding shaded areas represent the mean ± S.D.

As shown in Figure 3a,b, HADA exhibited preferential insertion at division sites and displayed a stronger HADA signal in the lateral wall in WT compared to the Δ*prc* cultured in LB medium lacking NaCl at 42 °C. This suggests that the absence of Prc reduces the activity of transpeptidases in LB medium lacking NaCl at 42 °C.

### 2.4. The Overabundance of MepS Contributes to the Growth Defect Observed in the Δprc

The Δ*prc* mutant exhibited a growth defect when cultured in LB medium without NaCl at 42 °C (Figure 3d). In addition to enzymes involved in peptidoglycan synthesis that may be regulated by NlpI-Prc, many hydrolases have been demonstrated to be negatively regulated by NlpI-Prc [27,28,30,32]. To investigate whether the overabundance of hydrolases in the Δ*prc* strain is responsible for its growth cessation, we conducted growth curve measurements in multiple knockout strains. The growth defect observed in the Δ*prc* mutant in LB medium without NaCl at 42 °C was alleviated by the absence of MepS (Figure 3d). The growth cessation observed in the Δ*nlpI-prc* mutant cultured in LB medium without NaCl at 42 °C could also be mitigated by the absence of MepS (Figure S2). To identify whether other hydrolases could have an additive alleviative effect, additional hydrolases were knocked out in the Δ*nlpI prc mepS* mutant. The absence of MltD in the Δ*nlpI prc mepS* mutant appeared to partially alleviate its growth defect in LB medium without NaCl at 42 °C (Figure S2).

### 2.5. Prc Negatively Regulates the Endopeptidase PBP7, but Not Endopeptidase PBP4

As shown in Figure 1b, the abundance of PBP7 and PBP5 in the Δ*nlpI-prc* mutant exhibited a significant increase and a slight increase, respectively, compared to the wild-type strain. In order to further investigate the potential negative regulation of PBP7 and PBP5 by NlpI-Prc, an in vivo degradation assay was performed. Strains were cultured in LB medium at 37 °C until reaching the exponential phase, and new protein synthesis was subsequently inhibited by spectinomycin (spec). The abundance changes in existing proteins in cells over time can be tracked by immunoblot. After addition of 400 μg/mL spec, samples were collected at indicated time points and the abundance changes in PBP7 in different strains were analysed by immunoblot.

**Figure 4:**
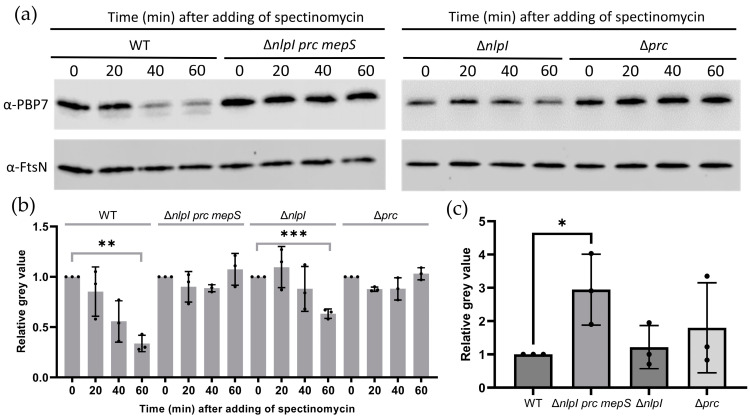
PBP7 can be degraded by Prc in an NlpI-independent manner. WT, Δ*nlpI*, Δ*prc,* and Δ*nlpI prc mepS* were cultured in LB medium at 37 °C, at an OD_600_ of ≈0.6, 400 μg/mL spec was added to block new proteins synthesis and samples were collected at indicated time points: (**a**) PBP7 and FtsN were detected using immunoblot analysis with specific antibodies against PBP7 and FtsN, respectively. FtsN was used as a loading control. The experiment was performed three times, and representative images are presented; (**b**) The normalized amount of PBP7 at indicated time points in different strains. The grey value of each strain at 0 min was set as 1 (control), and the change in grey value at each time point was plotted for each strain. Significance determined using an unpaired *t* test, was indicated as, ** *p* ≤ 0.01, *** *p* ≤ 0.001; and (**c**) The normalized amount of PBP7 in different strains in the absence of spec. The grey value of WT strain was set as 1 (control), and the relative grey value was plotted for each strain. Significance determined using an unpaired *t* test, was indicated as, * *p* ≤ 0.05.

In WT and Δ*nlpI* strains, a clear decrease in the amount of PBP7 was observed over time within an hour after the adding of spec (Figure 4a,b), suggesting that the existing PBP7 in the cells was degrading. Under the same conditions, the amount of PBP7 protein did not show noticeably decrease in the Δ*prc* and Δ*nlpI prc mepS* mutants (Figure 4a,b). This indicates that PBP7 is a substrate of the protease Prc and can be degraded by Prc in an NlpI-independent manner. The total amount of PBP7 in the absence of spec in the mutants did not show a significant difference compared to the wild-type strain, except for the Δ*nlpI prc mepS* where it was increased (Figure 4c).

In contrast, the amount of PBP5 did not show changes in the in vivo degradation assay (Figure S4). The interaction between NlpI and PBP4 was recently reported [32]. In order to verify whether PBP4 is also a target of Prc, the abundance of PBP4 after the addition of spec was also analysed by the in vivo degradation assay. As shown in Figure S3, the abundance of PBP4 remained unchanged within the first hour after the addition of spec in all strains.

### 2.6. Absence of NlpI-Prc Alleviates the Synthetic Lethality of the mepS mepM mepH Mutant Strain

Among the eight endopeptidases in *E. coli*, it has been experimentally shown that MepS, MepM, and MepH are redundantly essential for the bacterial growth and viability [25]. The mutant lacking these three PG hydrolases targeting the D-Ala-meso-DAP crosslink fails to incorporate new PG, leading to cell lysis [25]. The simultaneous absence of both MepS and MepM has been consistently demonstrated to be lethal in LB medium [27,50,51]. A recent study showed that the absence of NlpI or Prc can suppress the lethality of the *mepS mepM* mutant with an essential role played by MepH [27]. It was suggested that MepH has an essential function in this process. The findings reconfirmed the redundant essentiality of MepS, MepM, and MepH, and implies that MepH might be regulated by the NlpI-Prc complex. 

**Figure 5:**
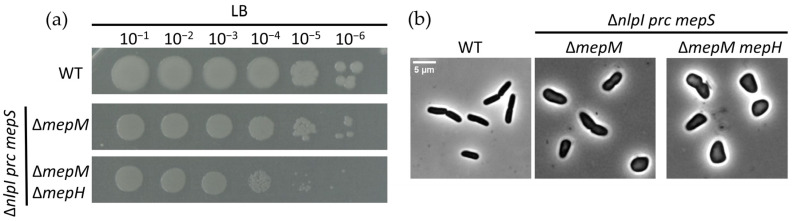
The absence NlpI-Prc alleviates the lethality of the *mepS mepM mepH* mutant strain. (**a**) Spot dilution assay on LB agar for WT, Δ*nlpI prc mepS mepM* and Δ*nlpI prc mepS mepM mepH.* Viability of WT cells in LB agar serves as a control for the mutants and (**b**) Phase-contrast images for the different strains cultured in LB medium at 37 °C. The scale bar equals 5 µm.

In order to explore the potential contribution of other hydrolytic enzymes to PG expansion and their regulation by the NlpI-Prc protein complex, the genes *mepS mepM* and *mepH* were successfully deleted in the Δ*nlpI-prc* double mutant using λ Red genome engineering (Figure 5a,b). The survival of the Δ*mepS mepM mepH nlpI prc* mutant indicates the involvement of other hydrolytic enzymes in PG expansion, which are probably under negative regulation by NlpI-Prc.

### 2.7. The Suppression of EDTA Sensitivity in the mepS Mutant Necessitates the Inactivation of NlpI-Prc, along with the Involvement of MepH, MltA and MltD

**Figure 6:**
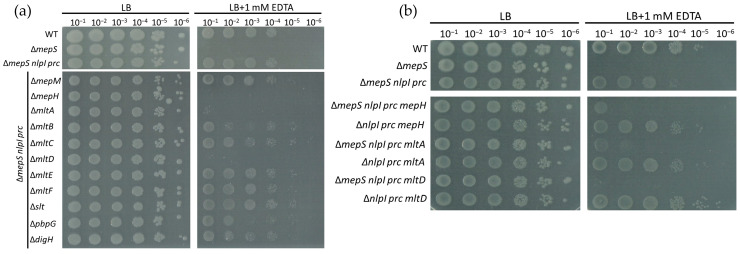
The absence of NlpI-Prc in Δ*mepS* suppresses its sensitivity to EDTA and the absence of MepH, MltA, or MltD in the Δ*mepS nlpI prc* restored the strains to high sensitivity to EDTA. (**a**,**b**) Strains cultured in LB medium at 37 °C for overnight were serially 10-fold diluted from 10^−1^ to 10^−6^ before spotting them onto LB plate and LB plate containing 1 mM EDTA. The viability of WT cells cultured in LB with 1 mM EDTA agar serves as a positive control for mutants cultured in same condition. The viability of strains cultured in LB agar serves as a cell loading control.

To investigate the involvement of other hydrolytic enzymes in PG expansion and their regulation by NlpI-Prc, we have streamlined the process of constructing new mutants. The additional hydrolytic enzymes were knocked out in the Δ*mepS nlpI prc* strain, rather than the Δ*mepS mepM mepH nlpI prc* strain. The Δ*mepS* has been demonstrated to be highly sensitive to EDTA [52]. In our study, we found that the Δ*mepS nlpI prc* mutant did not exhibit a difference in EDTA sensitivity compared to the wild-type strain (Figure 6a). By introducing an additional mutation into the Δ*mepS nlpI prc* strain, we can screen for the protein responsible for the reappearance of EDTA sensitivity. This protein may compensate for the absence of MepS and could be under the regulation of NlpI-Prc.

Knocking out *mepH*, *mltA*, or *mltD* in the Δ*mepS nlpI prc* strain restored the strains to high sensitivity to EDTA (Figure 6a). Implying that these three enzymes are possibly involved in the PG expansion and might be negatively regulated by NlpI-Prc. To rule out the possibility that the Δ*nlpI prc* mutant strain already exhibited sensitivity to EDTA when one of the three enzymes (MepH, MltA, or MltD) was absent, the *mepH*, *mltA*, or *mltD* genes were deleted in the Δ*nlpI prc* mutant background. As shown in Figure 6b, the newly constructed mutants in the Δ*nlpI prc* background did not exhibit increased sensitivity to EDTA.

### 2.8. PG Lytic Transglycosylase, MltD Is Negatively Regulated by NlpI-Prc

**Figure 7:**
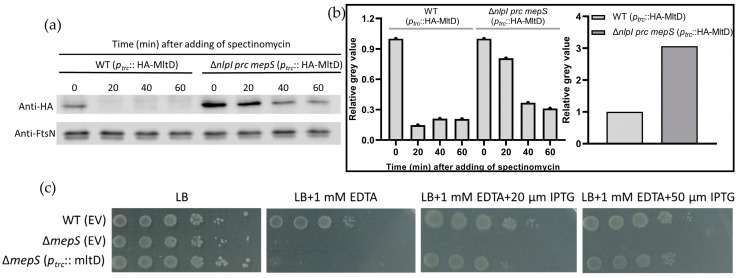
MltD is a substrate of the NlpI-Prc complex and complements the EDTA sensitivity of Δ*mepS*: (**a**) WT and Δ*nlpI prc mepS* harbouring a plasmid expressing HA-MltD were cultured in LB medium at 37 °C, when the OD_600_ reached 0.3, 400 μg/mL spec was added to block new proteins synthesis, and samples were collected at indicated time points. HA-MltD and FtsN were detected using immunoblot analysis with specific antibodies against the HA tag and FtsN, respectively. FtsN was used as a loading control and (**b**) The normalized amount of HA-MltD at indicated time points in different strains. Left panel: the grey value of each strain at 0 min was set as 1 (control) and the change in grey value at each time point was plotted for each strain. Right panel: the grey value of WT strain was set as 1 (as a control), and the relative grey value was plotted for Δ*nlpI prc mepS*. (**c**) The WT and *mepS* mutant cells harbouring the empty vector (EV) or vector expressing wild-type MltD were serially 10-fold diluted from 10^−1^ to 10^−6^ before spotting them onto LB plate and LB plate containing 1 mM EDTA. The viability of WT (EV) serves as a positive control. The viability of Δ*mepS* (EV) serves as a negative control.

The function of MepH in PG expansion and its negative regulation by NlpI-Prc have been previously described [27]. To investigate whether MltA and MltD are also regulated by NlpI-Prc, the HA tag was tried to fuse to the N-terminus of MltA and MltD on the plasmid. The abundance changes in fused proteins can be detected by antibodies against the HA tag. Unfortunately, the construction of the plasmid for the expression of HA-MltA was unsuccessful, possibly due to the toxicity of HA-MltA in cells. The fused protein HA-MltD was successfully constructed and expressed by the plasmid pXWL058 (Figure 7a). The original promotor and signal sequence of MltD were replaced by an IPTG-inducible promotor (*Ptrc* promotor) and the cleavable DsbA signal sequence (Dsba^ss^), respectively. The fused protein is transported into the periplasm by the cleavable Dsba^ss^ [53]. The fused HA-MltD appeared functional since it showed extreme toxicity in the Δ*prc* mutant (Figure S5).

The pXWL058 plasmid expressing HA-MltD was introduced into both WT and Δ*nlpI prc mepS* strains, and the stability of HA-MltD was evaluated using the in vivo degradation assay. As shown in Figure 7a,b, the abundance of HA-MltD (Time 0) in the WT strain was lower compared to that in the Δ*nlpI prc mepS* strain. The differential abundance of HA-MltD (Time 0) between the WT and Δ*nlpI prc mepS* strains could be attributed to the degradation activity of NlpI-Prc in WT. However, it is important to consider that the differential abundance of HA-MltD between the WT and Δ*nlpI prc mepS* strains could also be influenced by differences in the expression levels of the *HA-mltD* gene.

In the in vivo degradation assay, the degradation of HA-MltD in the Δ*nlpI prc mepS* strain is much slower compared to that in WT (Figure 7a,b). This suggests that HA-MltD is a substrate of NlpI-Prc. Our results are in agreement with the findings of Kaul et al., who also demonstrated that MltD is a substrate of the NlpI-Prc proteolytic system [50]. The slow degradation of HA-MltD despite the absence of Prc in the Δ*nlpI prc mepS* strain may be attributed to the action of other proteases.

The Δ*mepS* strain exhibits high sensitivity to EDTA, whereas the absence of NlpI-Prc in the Δ*mepS* strain alleviates this sensitivity. To further prove the MltD function and relationship between MltD and NlpI-Prc and avoid the potential toxicity of fused HA-MltD, wild-type MltD was expressed on a new plasmid (pAG003) in the Δ*mepS* strain. The expression of wild-type MltD is under control of the same IPTG-inducible promotor (*Ptrc* promotor). As shown in Figure 7c, the overexpression of MltD could complement the EDTA sensitivity of the *mepS* mutant. This suggests that the function of MepS can be substituted by the overexpression of MltD, further supporting the hypothesis that MltD is another substrate of the NlpI-Prc proteolytic system.

## 3. Discussion

PG expansion requires both PG synthesis and PG hydrolysis. One of the functions of hydrolysis is to create enough space for the insertion of nascent PG. In Gram-positive and Gram-negative bacteria, approximately 40–50% of the PG is degraded per generation by hydrolases. In order to prevent bacterial lysis due to excessive degradation of PG, the synthesis and degradation of PG during bacterial PG expansion are tightly regulated. The NlpI-Prc complex has been reported to be involved in this crucial regulatory process. However, the specific mechanisms by which the NlpI-Prc complex mediates PG synthesis enzymes and PG degradation enzymes are still largely unknown.

The absence of NlpI results in cell filamentation when cultured in hypotonic media at high temperatures [42]. Δ*prc* is unable to grow under the same conditions [33]. NlpI protects PBP3 from cleavage by Prc [45]. Suggesting that the NlpI-Prc complex might mediate important proteins that are involved in PG synthesis. PBP2 and PBP3 are essential, and PBP1A and PBP1B are semi-redundant essential for cell growth [10,17,54]. In our study, the abundance of PBPs involved in PG synthesis were assessed by Bocillin-binding assay and antibody against PBP3 in wild-type, Δ*nlpI,* and Δ*prc* cells. As shown in Figure 1b and Figure S1b,c, the abundance of PBPs in Δ*nlpI* and Δ*prc* mutants do not exhibit a noticeable decrease compared to wild-type cells, implying the growth defects in Δ*nlpI* and Δ*prc* mutants are not caused by the absence of some PBPs.

Except for the protein abundance, the subcellular localization of PBPs is vital for their functions. Compared to other important PBPs, PBP3 has the most pronounced subcellular localization characteristics and it is the essential transpeptidase in the divisome [17]. The Δ*nlpI* mutant cultured in LB medium without NaCl at 42 °C exhibited filamentous growth, that indicates the divisome is dysfunctional (Figure 1a). Active septal PG synthesis results in a strong HADA labelling at the cell division site. In the Δ*nlpI* strain cultured in LB medium without NaCl at 42 °C, the HADA labelling at the cell division site almost disappeared (Figure 2c). This also suggests that, under these culture conditions, the absence of NlpI leads to a dysfunctional divisome. In addition, we observed the absence of midcell localization of PBP3 in the Δ*nlpI* mutant under the same conditions (Figure 2a). The delocalized PBP3 in the Δ*nlpI* mutant might be the reason for its filamentous growth. However, it remains uncertain whether NlpI directly or indirectly influences the subcellular localization of PBP3, necessitating further validation.

In LB without NaCl medium at 42 °C, Δ*prc* stopped growing and the cells have a significant decline in the transpeptidase activity as monitored by the HADA labelling (Figure 3a,d). The stagnant growth in Δ*prc* indicates dysfunctional divisome and elongasome. Different from Δ*nlpI*, the reasons for the extra dysfunctional elongasome in Δ*prc* mutant require further research to explain it. As shown in Figure S1b,c, PBP3 exhibited a band of higher molecular weight in the Δ*prc* mutant compared to wild-type cells in which Prc cleaves 11 C-terminal amino acids of PBP3 [35]. However, the PBP3 and PBP3 without C-terminal 11 amino acids are both functional [33,34]. Whether the C-terminal 11 amino acids of PBP3 influence its substrate binding or protein–protein interaction needs further exploration.

Except for the PG synthesis changes in Δ*prc* mutant, we hypothesize that the overabundance of hydrolyses and lytic transglycosylases could be the reason for the growth arrest in Δ*prc* mutant. By employing multiple deletions in the Δ*prc* mutant or Δ*nlpI prc mepS* mutant strain, we found that MepS contributes to the growth defect observed in Δ*prc*, and MltD also moderately constrains the growth of the Δ*nlpI prc mepS* mutant (Figure 3d and Figure S2). The disorder between PG synthesis and degradation might be part of the reason for the growth defect in Δ*prc* mutant.

The reasons for the filamentous growth of Δ*nlpI* and the growth stagnation of Δ*prc* in LB medium without NaCl at 42 °C have yet to be fully determined. While the possibility that reduced abundance of PBPs in these mutants is responsible for their growth defect has been ruled out, we still lack information about changes in the abundance of other essential enzymes for PG synthesis, such as RodA and FtsW, within these mutants. Even though the compromised midcell localization of PBP3 was observed in the Δ*nlpI* mutant cultured in LB medium without NaCl at 42 °C, the reasons for the dysfunctional divisome were not disclosed. The absence of some hydrolyses relieved the viability pressure of the Δ*prc* mutant cultured in LB medium without NaCl at 42 °C. However, this may only be part of the reason for the cessation of growth in the Δ*prc* mutant under these conditions. In addition, the NlpI-Prc was reported to be involved in the mediation between the outer membrane and peptidoglycan growth [55]. Given that the NlpI-Prc complex interacts with numerous proteins, the filamentous growth of Δ*nlpI* and cessation of growth observed in the Δ*prc* mutant likely result from multiple factors.

The role of the NlpI-Prc complex in mediating peptidoglycan hydrolytic enzymes is better understood compared to its mediation of proteins involved in peptidoglycan synthesis. Many endopeptidases, such as MepS, MepM, and MepH, have been reported to be negatively regulated by the NlpI-Prc complex. In our research, we discovered that PBP7 is another substrate of the Prc protease, and its degradation by Prc occurs independently of NlpI (Figure 4). The absence of NlpI-Prc can alleviate the synthetic lethality observed in the *mepS mepM mepH* mutant strain (Figure 5). Because MepS, MepM, and MepH were identified as redundant essential “space makers”, the survival of the Δ*mepS mepM mepH nlpI prc* mutant indicates the existence of other “space makers” in PG expansion that might be mediated by NlpI-Prc complex. Through multiple deletions, we discovered that lytic transglycosylases such as MltA and MltD might fulfil this function (Figure 6a). Furthermore, it was found that the MltD is a substrate of the NlpI-Prc complex and the function of MepS can be substituted by the overexpression of MltD (Figure 7).

Understanding the mechanisms by which these degradation and modification enzymes are regulated and coordinated with PG synthesis is a major focus in the field. Various enzymes were shown to be degraded by NlpI-Prc proteolytic complex or degraded by Prc in an NlpI-independent manner, including endopeptidases (MepS and MepH), lytic transglycosylases (MltB, DigH, and MltG) [27,28,30,31]. This study demonstrated the proof that PBP7 and MltD are new substrates of the NlpI-Prc proteolytic complex. 

The activity and subcellular localization changes in some PG transpeptidases in the Δ*nlpI* and Δ*prc* are affected. The dysfunctional divisome in the Δ*nlpI* mutant might is the reason for its filamentous growth. Furthermore, delocalization of PBP3 in the Δ*nlpI* mutant has been confirmed, the mechanisms through which NlpI influences PBP3 assembly within the divisome require further investigation. The cessation of growth observed in the Δ*prc* mutant is likely cause by multiple factors. The absence of MepS in the Δ*prc* mutant relieved partly its growth defect. The growth stagnation observed in the Δ*prc* mutant may be attributed to the imbalance between PG synthesis and hydrolysis. In addition, a novel function of the NlpI-Prc complex in mediation growth between the outer membrane and peptidoglycan was recently proposed [55]. The NlpI-Prc complex interacts with numerous proteins involved in PG synthesis, PG degradation and outer membrane synthesis. The crucial role of the NlpI-Prc complex in cell envelope growth is self-evident but remains less understood. Further work is needed to explore how NlpI-Prc is involved in coordinating peptidoglycan synthesis and degradation, and in balancing peptidoglycan expansion and outer membrane synthesis.

## 4. Materials and Methods

### 4.1. Bacterial Strains and Culture Conditions

*E. coli* strains used in this work are listed in Supplementary Table S1. All *E. coli* strains used in this work are derivatives of BW25113. Strains were cultured in LB medium (10 g Tryptone (Duchefa, Haarlem, The Netherlands), 5 g yeast extract (Fisher Bioreagents, Pittsburgh, PA, USA), and 10 g NaCl (Acros Organics, Geel, Belgium) per litre at 37 °C or in LB medium without NaCl at 42 °C. Strains were cultured in minimal glucose (GB4) medium (6.33 g K_2_HPO_4_·3H_2_O (VWR International, Radnor, PA, USA), 2.95 g KH_2_PO_4_ (Fisher Chemical, Waltham, MA, USA), 1.05 g (NH_4_)_2_SO_4_ (Sigma-Aldrich, Burlington, MA, USA), 0.10 g MgSO_4_·7H_2_O (Roth, Karlsruhe, Germany), 0.28 mg FeSO_4_·7H_2_O (Sigma-Aldrich, Burlington, MA, USA), 7.1 mg Ca (NO_3_)_2_·4H_2_O (Sigma-Aldrich, Burlington, MA, USA), 4 mg thiamine (Sigma-Aldrich, Burlington, MA, USA), 2 mg uracil (Sigma-Aldrich, Burlington, MA, USA), 2 mg lysine (Sigma-Aldrich, Burlington, MA, USA), 2 mg thymine (Sigm-Aldrich, Burlington, MA, USA), and 0.5% glucose (Roth, Karlsruhe, Germany), per litre, pH 7.0) at 28 °C. When necessary, the following antibiotics were introduced to the medium: 25 µg/mL chloramphenicol (Sigma-Aldrich, Burlington, MA, USA), 50 µg/mL kanamycin (Sigma-Aldrich, Burlington, MA, USA), 10 µg/mL tetracycline (Sigma-Aldrich, Burlington, MA, USA), 400 μg/mL spectinomycin (Duchefa, Haarlem, The Netherlands), and 100 µg/mL ampicillin (Roth, Karlsruhe, Germany).

### 4.2. E. coli Deletion Strains and Plasmids Construction

*E. coli* deletion strains were constructed by λ-Red recombination as described previously [56]. The primers used for the construction of deletion strains are listed in Supplementary Table S2. After PCR products size checking, the products were electroporated into BW25113 harbouring the plasmid pKD46. The PKD46 carries a gene encoding red recombination protein, which facilitates homologous recombination between the linear DNA fragment with the *E. coli* genome at the regions of homology. The red recombination protein was induced with 0.2% L-arabinose (Sigma-Aldrich, Burlington, MA, USA). Recombinants were selected on LB plates containing 25 µg/mL chloramphenicol or 50 µg/mL kanamycin. If required, the resistance gene in the genome were removed by temperature-sensitive plasmid pCP 20. For plasmids construction, all primers used in this work are listed in the Supplementary Table S2. The plasmids used in this paper are listed in the Supplementary Table S3. Following the PCR purification, the PCR products were digested by DpnI and were verified by DNA agarose gel electrophoresis and DNA sequencing with the mix2seq kit (Eurofins, Luxembourg). The Gibson assembly method [57] was employed for the construction of new plasmids and the assembled products were directly transform into DH5α competent cells for plasmids storing and checking.

### 4.3. Bocillin-Binding Assay

The Bocillin-binding assay was carried out as described previously and modifications were made to the details [58]. Strains were cultured in LB medium overnight at 37 °C. The second day, overnight mediums were diluted 1:1000 in fresh LB medium and grown until an OD_600_ of ≈0.3. Cells were collected by centrifugation (8000× *g* for 2 min at room temperature). After washing with 1 mL PBS, the pellet was resuspended in 50 µL PBS containing 5 µg/mL Bocillin-FL (Thermo Fisher Scientific, Waltham, MA, USA) and incubated for 10 min. The pellets were washed 2 times with 100 µL PBS and then resuspended in 100 µL PBS to which 20 µL 5X Protein Loading Buffer (250 mM Tris buffer (VWR International, Radnor, PA, USA) at pH 8.3, 10% SDS (Merck, Darmstadt, Germany), 500 mM DTT (Sigma Aldrich, Burlington, MA, USA) and 50% Glycerol from Biosolve Chimie, Dieuze, France) was added. The samples were heated for 10 min at 99 °C to denature the proteins and 10 µL was loaded on a 10% SDS-PAGE gel. The gel was scanned using a LICOR Odyssey M Imager (LI-COR, Lincoln, NE, USA) at 520 nm.

### 4.4. HADA Labelling

A modified protocol of HADA labelling was carried out [47]. The strains in the exponential growth phase were harvested by centrifugation at 8000× *g* for 2 min and then resuspended in pre-warmed LB medium with and without NaCl, supplemented with 250 µM fluorescent D-amino acid HADA (Bio-Techne, Minneapolis, MN, USA), for a 10-min incubation at 37 °C and 42 °C. Cells were collected by centrifugation (8000× *g* for 2 min). The pellets were fixed in 70% ethanol (Merck, Darmstadt, Germany) for 10 min. After washing 2 times with PBS, samples were immobilized on an object glass slide coated with 1% agarose and imaged with an Olympus BX-60 (Olympus, Tokyo, Japan) equipped with a Hamamatsu ORCAFlash-4.0LT CMOS camera (Hamamatsu Photonics, Shizuoka, Japan) fluorescence microscope through a 100×/N.A. 1.35 oil objective. The filter used was U-MWU (Olympus, Tokyo, Japan) longpass (excitation at 330–385 nm, emission > 420 nm). Images were taken using the program ImageJ 1.53f (http://imagej.nih.gov/ij/, accessed on 25 October 2020) with MicroManager 1.4.22 (https://www.micro-manager.org, accessed on 14 August 2015).

### 4.5. Immunolabeling

The strains used for immunolabeling were cultured in GB4 medium to steady state at 28 °C and fixed by 2.8% formaldehyde (Sigma-Aldrich, Burlington, MA, USA) and 0.004% glutaraldehyde (Sigma-Aldrich, Burlington, MA, USA) for 15 min. The immunolabeling experiments were carried out as described before [59]. A rabbit polyclonal FtsN (1:500) antibody [60] and the second antibody Cy3-AffiniPure Donkey Anti-Rabbit IgG (1:300) (Jackson Immunochemistry, West Grove, PA, USA) were used. The immunolabeled cells were immobilized on 1% agarose slab and imaged with a BX-60 fluorescence microscope (Olympus, Tokyo, Japan). The mCherry filter (excitation at 560 ± 40 nm and emission at 630 ± 75 nm) was used in this experiment.

### 4.6. Spot Assay

To examine the sensitivity of *E. coli* strains to EDTA, cells were cultured overnight in LB medium at 37 °C. Cells were serially 10-fold diluted from 10^−1^ to 10^−6^ before loading 2 μL samples onto LB agar and LB agar containing 1 mM EDTA (Sigma-Aldrich, Burlington, MA, USA). The expression of wild-type MltD from pAG003 was induced with 20 or 50 µM isopropyl ß-D-1-thiogalactopyranoside (IPTG, Duchefa, Haarlem, The Netherlands). The LB agars were incubated overnight at 30 °C.

### 4.7. Immunoblotting

Samples were separated by SDS-PAGE and transferred onto nitrocellulose (Bio-Rad, Hercules, CA, USA) in a semi-dry transfer manner as described previously [61]. The membranes were blocked with 5% skimmed milk (Carl Roth, Karlsruhe, Germany) in TBS solution (20 mM Tris, 150 mM NaCl, adjusted pH to 7.5 by HCl) for 1 h and then incubated overnight with appropriate primary antibodies (1:2000 for α-PBP7, 1:5000 for α-HA (H6908, Sigma-Aldrich, Burlington, MA, USA), 1:0000 for α-FtsN [60], 1:1000 for α-PBP3 [46], 1:2000 for α-PBP4 [62], and 1:2000 for α-PBP5 [63] at 4 °C. Membranes were washed three times with TBST solution (TBS containing 0.01% Tween-20 from Sigma-Aldrich, Burlington, MA, USA) and incubated with secondary antibodies (1:5000) tagged with horseradish peroxidase (HRP) (SAB3700863, Sigma-Aldrich, Burlington, MA, USA) for one hour at room temperature. The chemical signal of HPR were detected by ECL Prime detection substrate (32109, Thermo Fisher Scientific, Waltham, MA, USA).

### 4.8. Image Analysis

The phase-contrast and fluorescence images, captured using the ImageJ 1.53f program (http://imagej.nih.gov/ij/, accessed on 25 October 2020), were merged into hyperstacks and linked to the Coli-Inspector project file, running in conjunction with the ObjectJ-1.05n plugin (https://sils.fnwi.uva.nl/bcb/objectj/, accessed on 24 May 2022). Each cell in the images can be delineated, and background fluorescence can be subtracted before analysis. Image analysis was carried out as previously described [64]. Based on the data, cells can be sorted by their length, and the fluorescence intensity corresponding to the local fluorescence of an individual cell can be represented in demographs, with each cell represented as a line.

### 4.9. Statistical Analysis

For the HADA labelling and in vivo degradation assay of PBP7, *p*-values were calculated with unpaired two-tailed *t*-tests using Prism software 9.4.1 (GraphPad Software, San Diego, CA, USA). Statistical significance was indicated as follows: * *p* < 0.05; ** *p* ≤ 0.01; *** *p* ≤ 0.001, **** *p* ≤ 0.0001. *p*-values less than 0.05 were considered statistically significant.

## 5. Conclusions

The NlpI-Prc complex regulates the activity of PG transpeptidases and subcellular localization of PBP3. The NlpI-Prc complex negatively regulates PBP7 and MltD. The absence of NlpI-Prc complex can alleviate the lethality of the mepS mepM mepH mutant. Based on multiple gene deletions, we propose a function of the PG lytic transglycosylases MltA and MltD as “space makers”. It is clear that NlpI-Prc functions as a central hub for the regulation of the availability of a multitude of proteins involved in PG synthesis and degradation. The exact nature of how it functions requires further investigation.

## Data Availability

Data is contained within the article or supplementary material.

## Supplementary Materials

The following supporting information can be downloaded at: https://www.mdpi.com/article/10.3390/ijms242216355/s1. References [65,66] are cited in the supplementary materials.
