# Supplementary material for: NlpI-Prc Proteolytic Complex Mediates Peptidoglycan Synthesis and Degradation via Regulation of Hydrolases and Synthases in Escherichia coli
Source: Int J Mol Sci. 2023 Nov 15;24(22):16355. doi: 10.3390/ijms242216355 (PMC10671308; doi:10.3390/ijms242216355)
Supplement: Supplementary file 1 [file ijms-24-16355-s001.zip › ijms-2693653-supplementary.pdf]

Supplementary Material

NlpI-Prc proteolytic complex mediates peptidoglycan synthesis and degradation by regulation of hydrolases and synthases in *Escherichia coli*

Xinwei Liu and Tanneke den Blaauwen*

*** Correspondence: t.denblaauwen@uva.nl**

Supplementary Figures

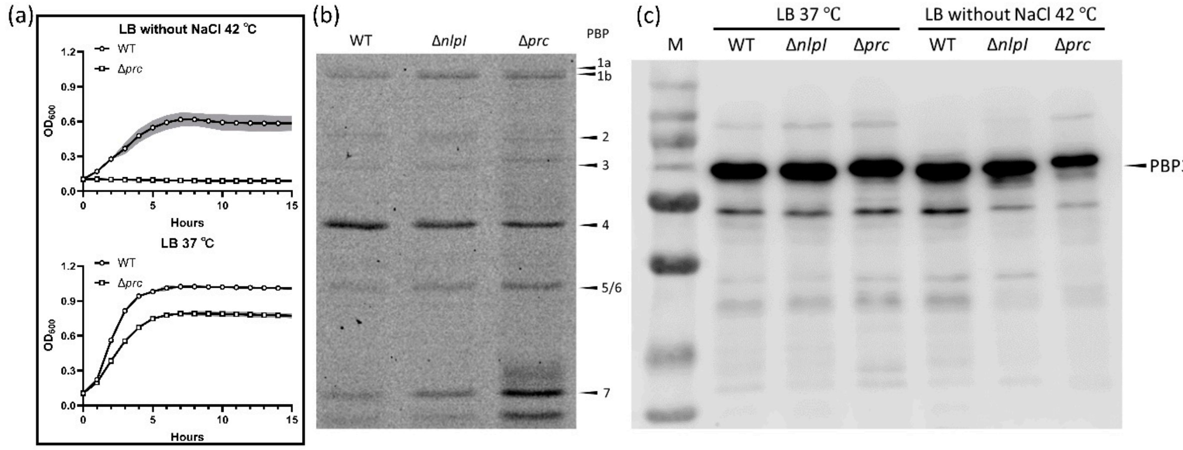

Figure S1 Growth curve and PBPs abundance of strains. (a) Strains were cultured in LB medium at 37 °C or in LB medium without NaCl at 42 °C for OD_{600} measurements. The solid lines and their corresponding shaded areas represent the mean ± S.D. (b) Bocillin-FL binding to PBPs in strains cultured in LB. (c) Immunoblot showing PBP3 abundance in WT, Δ*nlpI and* Δ*prc* strains cultured as above.

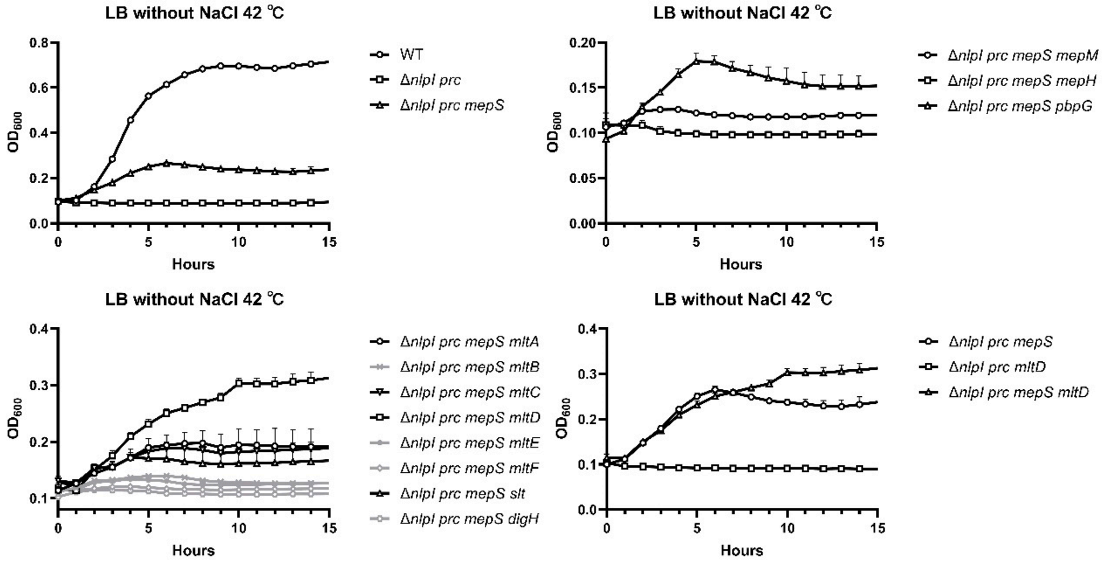

Figure S2 Growth curve of strains cultured in LB medium without NaCl at 42 °C. Growth curves were performed in triplicate for each mutant. Time in is plotted against the OD_{600}.

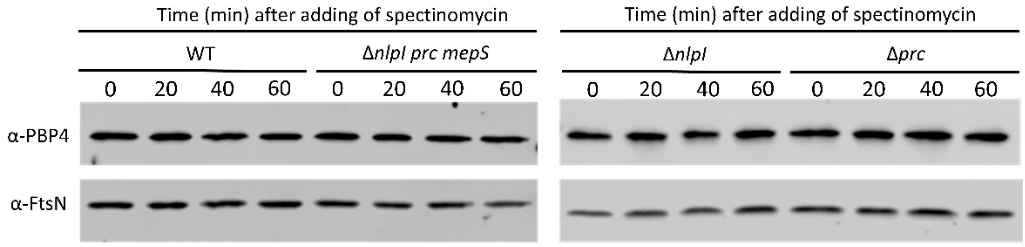

Figure S3 Stability of PBP4 determine by an *in vivo* degradation assay. Strains were cultured in LB at 37 °C until an OD_{600} of ≈ 0.3. Spec was added in medium to block translation, and samples were collected at indicated time points. PBP4 and FtsN were visualized using immunoblot analysis with specific antibodies against PBP7 and FtsN, respectively. FtsN was used as a loading control.

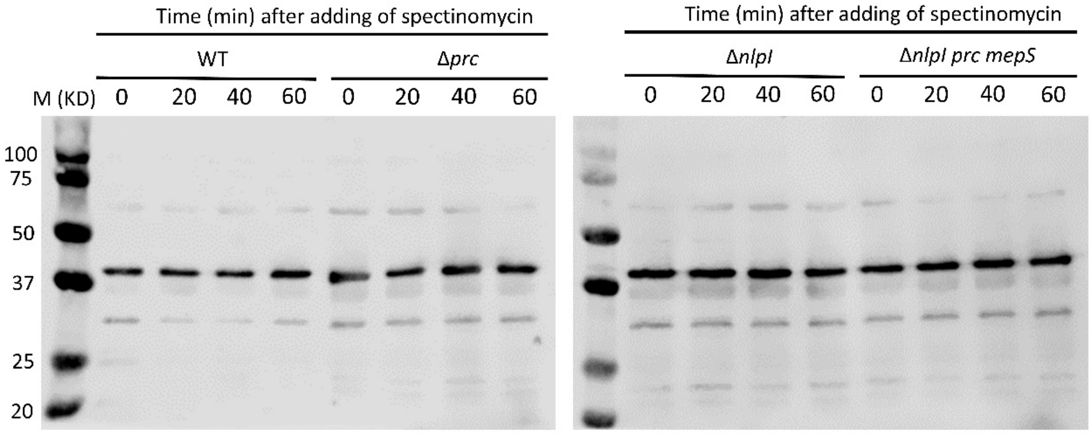

Figure S4 Stability of PBP5 determine by an *in vivo* degradation assay. Strains were cultured in LB at 37 °C until an OD_{600} of ≈ 0.3. Spec was added in medium to block translation, and samples were collected at indicated time points. PBP5 was visualized using immunoblot analysis with specific antibodies against PBP5.

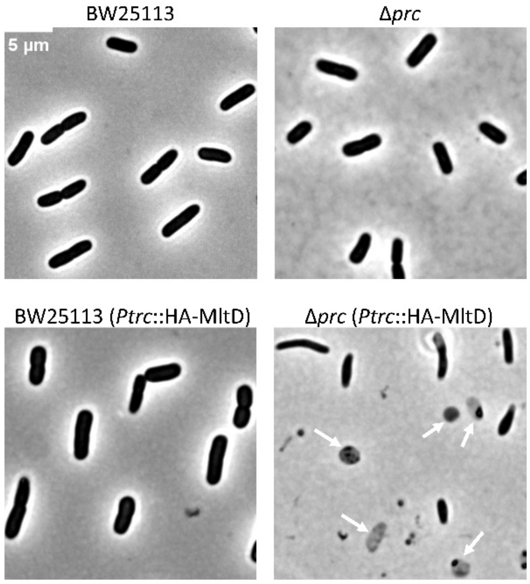

Figure S5 The fused HA-MltD protein showed extreme toxicity in the Δ*prc* mutant. The morphology of wild-type (BW25113), Δ*prc*, BW25113 carrying pXWL058-*Ptrc*::HA-MltD and Δ*prc* mutant carrying pXWL058-*Ptrc*::HA-MltD cells cultured in LB medium. The scale bar equals 5 µm. The lysed cells were pointed out by a white arrow.

Supplementary Tables

Table S1. Strains used in this work

Strain	Mutation	Genotype	Source
BW25113	Wild-type	*F-, Δ(araD-araB)567, lacZ4787(Δ)::rrnB-3, LAM-, rph-1, Δ (rhaD-rhaB)568, hsdR514*	*[33]*
	ΔnlpI	*ΔnlpI::tet*^{R}	*[14]*
	Δprc	*Δprc::cat*^{R}	This work
	ΔmepS	*ΔmepS:: tet*^{R}	This work
	ΔnlpI prc	*ΔnlpI::tet*^{R}*Δprc::cat*^{R}	This work
	ΔmepS prc	*Δprc mepS::kan*^{R}	This work
	ΔnlpI prc mepS	*ΔnlpI::tet*^{R} *ΔmepS::kan*^{R} *Δprc::cat*^{R}	This work
	ΔnlpI prc mepS mepM	*ΔnlpI prc mepS mepM::kan*^{R}	This work
	ΔnlpI prc mepS mepM	*ΔnlpI prc mepS*	This work
	ΔnlpI prc mepS mepH	*ΔnlpI prc mepS mepH::kan*^{R}	This work
	ΔnlpI prc mepS mltA	*ΔnlpI prc mepS mltA::kan*^{R}	This work
	ΔnlpI prc mepS mltB	*ΔnlpI prc mepS mltB::kan*^{R}	This work
	ΔnlpI prc mepS mltC	*ΔnlpI prc mepS mltC::kan*^{R}	This work
	ΔnlpI prc mepS mltD	*ΔnlpI prc mepS mltD::kan*^{R}	This work
	ΔnlpI prc mepS mltE	*ΔnlpI prc mepS mltE::kan*^{R}	This work
	ΔnlpI prc mepS mltF	*ΔnlpI prc mepS mltF::kan*^{R}	This work
	ΔnlpI prc mepS slt	*ΔnlpI prc mepS slt::kan*^{R}	This work
	ΔnlpI prc mepS pbpG	*ΔnlpI prc mepS pbpG::kan*^{R}	This work
	ΔnlpI prc mepS digH	*ΔnlpI prc mepS digH::kan*^{R}	This work
	ΔnlpI prc mepH	*ΔnlpI prc mepH::kan*^{R}	This work
	ΔnlpI prc mltA	*ΔnlpI prc mltA::kan*^{R}	This work
	ΔnlpI prc mltD	*ΔnlpI prc mltD::kan*^{R}	This work
DH5α	Storage strain	*F- endA1 glnV44 thi-1 recA1 φ80ΔlacZΔM15, λ*$^-$	Invitrogen

Table S2. Primers used in this work

Purposes	Name	Sequence 5′-3′
prc Knock out in genome	XW 142	ggcgtccgcgtccagctgaattc
	XW 143	gtagcatctgatttacggcatcttgtcgctg

mepM Knock out in genome	XW 107	attagccaaccagtatgcgagctgc
	XW 108	gcgcaacttgccccgcacca
mepH Knock out in genome	XW 226	gcaggcatgatagacctgcctttacagagggacgctcagtgtaggctggagctgcttc
	XW 227	atgaacttaccctgttgccgtaacaacagggtaaagttatgggaattagccatggtcc
mepH check in genome	XW 137	ttgtagcgggtcagtgcggcatc
	XW 138	gcatacgtacaatagccttattgtgc
mltA knock out in genome	XW 235	ttcgttgcgccttattttttaacctgaagaagagaacagtgtaggctggagctgcttc
	XW 236	cctgtcatatccgtaaaaacggcatacagaatatcacaatgggaattagccatggtcc
mltA check in genome	XW 237	ggatgcgcactatactccgctc
	XW 238	caggatgtgaggttacccctcac
mltB knock out in genome	XW 239	atacttgcccctggttgaatctgttaaatggacccctcgtgtaggctggagctgcttc
	XW 240	tagccagagggaagctcacgccccccctcttgtaaatagatgggaattagccatggtcc
mltB check in genome	XW 241	aagtccgtatcgggcagtgac
	XW 242	accaccggtacaagactcggc
mltC knock out in genome	XW 243	aacttgcataaaaacaaacacaacacgcacccggaatggtgtaggctggagctgcttc
	XW 244	tttttgcccctgagcatcgtcaggggcggttaatggaaatgggaattagccatggtcc
mltC check in genome	XW 245	aggagatggtcaacttcctgttc
	XW 246	ggattttcagctgcagcttaagattc
mltD knock out in genome	XW 228	tatgatcggtcgtcttttaagcaactattgacacacacgtgtaggctggagctgcttc
	XW 229	gcaccgggggaatcggtgccttttttattatctggtttgatgggaattagccatggtcc
mltD check in genome	XW 230	aacaacctgaagagcgttttgc
	XW 231	tggtagatgcacataaagcggc
mltE knock out in genome	XW 247	cacctcaacggcgattccaggctataaggatagaagaagtgtaggctggagctgcttc
	XW 248	agaagcccgggaaaaagcggacaaagtgcgcgactgatatgggaattagccatggtcc
mltE check in genome	XW 249	tcggtaaggcgctggatacc
	XW 250	tctctctcgagcgagaagccc
mltF knock out in genome	XW 251	ttcaagctgggacgcgcacgacacagagaattaactaagtgtaggctggagctgcttc
	XW 252	gaaattaaagcgcagaaaaaaagcgcaatcctcgacggaatgggaattagccatggtcc
mltF check in genome	XW 253	gtgaattagcaacgcgtgcc
	XW 254	gttgctcagtgacttctttcgc
slt knock out in genome	XW 167	aacaactttgccgacacgatgc
	XW 168	tggtgacgctgttctgccatc

digH knock out in genome	XW 131	gagaatgagcgcacatctgtttaccggaaaccagcacatgtgtaggctggagctgcttc
	XW 132	gggtaaagcaccggcttgttacaaagtaagaatgggagtatgggaattagccatggtcc
digH check in genome	XW 133	cgctcccttttcgtgcttgctgtc
	XW 134	acgctactgttcttcagaagagtatagccc
pbpG knock out in genome	XW KO PBP7-F	ccggcggtgcgcaacccgtgcgcgtgaaccactatctgagtgtaggctggagctgcttc
	XW KO PBP7-R	aaaaattacggatggcagagtatcgccatccgaattcacatgggaattagccatggtcc
pbpG check in genome	XW P-pbpG C-F	gcactcccgttctggataatg
	XW P-pbpG C-F	ttatcagaccgcttctgcg
pXWL058 Construction	XW 232	cagaacctccagcgtaatctggaacatcgtatgggtacagaccagccggaccctcgag
	XW 233	acgctggaggttctggtggatcatgccagagtaccggcaacgt
	XW 272	tcaggaatctggcatgttgttgtttttc
	XW 273	tgccagattcctgaaaagaagcttggctgttttggcg
pAG003 Construction	prAAFG005	ttaccatggcaaaggcaaaagcgatattactcgcctctg
	prAAFG006	tttaagcttcaggaatctggcatgttgttgtttttcac
Plasmid insert check	XW 31	gcataattcgtgtcgctcaaggc
	XW 34	gttttatcagaccgcttctgcgtt

Table S3. Plasmids used in this work

**Name**	**Characteristics**	**Source**
PKD3	contain the FRT-CAM-FRT cassette for gene inactivation	*[56]*
PKD4	contain the FRT-KAN-FRT cassette for gene inactivation	*[56]*
PKD46	Contain λ-phage red recombinase proteins induced by arabinose	*[56]*
pCP20	Thermal induction of FLP synthesis	*[56]*
pTHV037	Basic expression vector, *Ptrc* promoter, pBR322 origin and ampR	*[65]*
pSAV057	Basic expression vector, *Ptrc* promoter, p15 origin and catR	*[66]*
pXWL058	pSAV057-DsbA^{SS}-HA-MltD	This work
pAG003	pSAV057-MltD	This work
